# Cell macroencapsulation devices in contemporary research: A systematic review

**DOI:** 10.1016/j.reth.2025.05.013

**Published:** 2025-06-09

**Authors:** Murillo D.L. Bernardi, Natascha Rus, Robin W.M. Vernooij, Marianne C. Verhaar, Maarten B. Rookmaaker

**Affiliations:** a University Medical Center Utrecht, Department of Nephrology, Utrecht, the Netherlands; b Independent Researcher, the Netherlands

**Keywords:** Cell encapsulation, Bioencapsulation, Biomedical devices, Implantation, Therapeutic delivery

## Abstract

Cell macroencapsulation devices (CMDs) represent a promising therapeutic approach by combining controlled, off-the-shelf technology with living tissue functionality. Despite their potential, the current knowledge about CMD development and implementation remains fragmented across different applications. We conducted a systematic review to synthesize the available evidence on implantable CMDs and establish a comprehensive framework for their development. Our analysis cataloged studies based on device materials and design, cell types, implantation strategies, and evaluation methods. The review revealed that CMD development has largely progressed in application-specific silos, with varying approaches to device characterization, cell selection (from xenogeneic to autologous), and assessment methods. Key evaluation parameters included cell survival and functionality, host tissue response (inflammation, vascularization, and fibrosis), and therapeutic efficacy. While successful implementations exist across multiple applications, the lack of standardized development and evaluation protocols emerges as a significant barrier to cross-application advancement. These findings highlight the need for unified assessment frameworks to accelerate CMD development across therapeutic applications.

## 1 Introduction

Progressive organ failure is a growing problem with aging population. While advancements in medication and technical devices have offered some solace, their limitations in mimicking the complex interplay of living tissues are becoming increasingly apparent. Regenerative cell-based therapies promise a paradigm shift in our approach to organ dysfunction. However, hurdles like shortage of donor cells, tissue and organs, limited cell survival after transplantation, the risk of malignant degeneration and immune rejection continue to impede their widespread adoption [[1], [2], [3], [4]].

Cell Macroencapsulation Devices (CMD) emerge as a promising approach in this complex landscape. These devices, which encapsulate cells within a protective membrane, offer a unique blend of off-the-shelf accessibility, immune protection while preventing malignant infiltration, and even the potential for removal if needed [5]. Unlike their microencapsulated counterparts, CMDs offer greater cell capacity, mechanical stability, and improved immune protection, enabling more effective and durable therapeutic outcomes [6]. In addition, control over the macro and meso structure of the device provides the opportunity to enhance differentiation and functionalization. However, the absence of standardized design protocols and testing frameworks limits the comparability of studies and hinders progress in optimizing CMD efficacy.

Unlocking the true potential of CMDs requires a comprehensive analysis of key factors influencing their effectiveness. This systematic review dissects three crucial areas: (i) device characteristics, including shape, material, and cell source, (ii) implantation characteristics, such as animal models, site, and duration, and (iii) functional outcomes, focusing on tissue responses like fibrotic and vascularizing reactions, as well as the viability and functionality of the encapsulated cells. Here, we compile current research on CMDs, systematically outlining these aspects as covered in available studies. This structured examination of historical design and assessment strategies not only aids in advancing existing CMDs but also sparks ideas for novel applications of this promising technology.

## 2 Methods

To ensure a thorough and systematic approach, a detailed search strategy was developed and implemented across two major databases, PubMed and Embase. The search strategy combined a range of keywords and MeSH terms relevant to cell encapsulation, implantation, and specific devices, returning 413 results from PubMed and 279 from Embase (Supplemental Table 1). After removing 156 duplicates, 539 unique studies were identified for screening.

Two independent reviewers conducted an initial screening of titles and abstracts based on predefined exclusion criteria: studies not being original research, not involving cell macroencapsulation devices, not being animal studies, not written in English, or not being accessible. This rigorous screening process ensured that only relevant studies proceeded to the data extraction phase.

For data extraction, the same two reviewers independently extracted key information from each included study to maintain accuracy and consistency. The extracted data included general study characteristics and details about the CMDs, including materials used, design types, and fabrication methods. Implantation characteristics, such as implantation sites and animal models, were also recorded, alongside ethical considerations and functional outcomes. Functional outcomes focused on the types of cells encapsulated, the therapeutic applications explored, and specific readouts like fibrotic response and vascularization.

To synthesize the data, descriptive statistics were employed to summarize findings and analyze trends over four distinct time periods: 1980–1990, 1991–2000, 2001–2010, and 2011–2024. Additionally, ethical considerations were meticulously noted, with explicit mentions of adherence to animal welfare guidelines and ethical approval, reflecting an increasing emphasis on ethical standards in recent studies.

## 3 Results

### 3.1 Literature search and selection

**Fig. 1:**
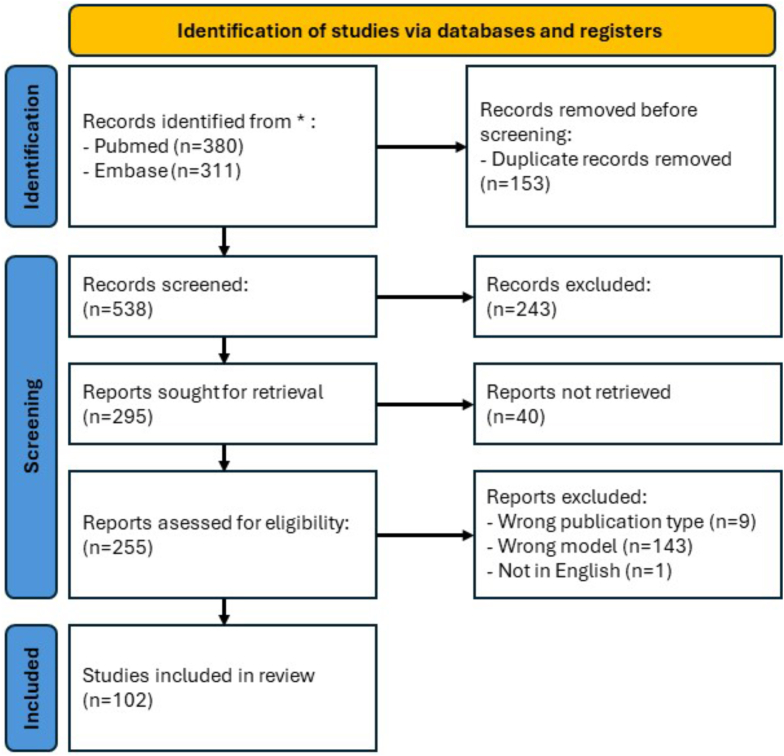
**Search parameters and inclusion criteria for studies in this review**.

Our systematic search yielded 691 publications from two databases. After removing duplicates (*n* = 153), titles and abstracts of 538 articles were screened. Of these, 243 articles were excluded based on pre-defined eligibility criteria (Fig. 1). Following a thorough review of 295 full-text articles, a further 193 were excluded based on the detailed criteria outlined in Fig. 1. Ultimately, 102 studies were deemed eligible for qualitative synthesis, with publishing dates ranging from 1980 all the way into 2024.

### 3.2 Categorization of the studies

**Fig. 2:**
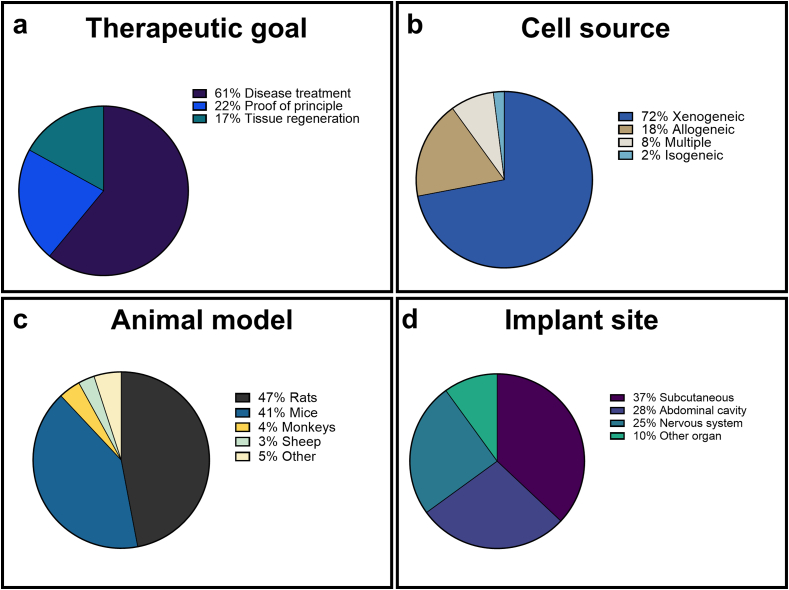
**Distribution of studies in the categories: therapeutic goals, cell source, animal model, and implantation site**.

The various therapeutic applications found in CMD research were categorized into three overarching goals based on their primary intended outcome (Fig. 2a).

- **Disease treatment (61 %):** In this category we identified studies specifically targeting the treatment or management of diagnosed medical conditions. This category includes applications where CMDs are used to deliver therapeutic molecules or cells to address specific pathologies, with diabetes being the most investigated disease (accounting for 42 % of all studies), followed by neurodegenerative diseases, such as Parkinson’s and Huntington’s (19 %). Examples include studies investigating the neuroprotective effects of encapsulated cells delivering glial cell line-derived neurotrophic factor (GDNF) to the brain [7,8], l-dopa-secreting cells for Parkinson’s disease [9,10], and Nerve Growth Factor (NGF) for Alzheimer’s disease [11,12]. CMDs were also explored for diverse conditions like arthritis, using interleukin-secreting cells [13] and cancer, with endostatin-secreting cells to inhibit angiogenesis [14,15].
- **Proof-of-principle studies (22 %):** Here we categorized studies where the primary objective is to validate new CMD designs, materials, or applications. These studies focus on demonstrating device functionality, such as establishing immunoprotection capabilities, testing novel membrane materials, or validating new encapsulation methods [[16], [17], [18], [19], [20], [21], [22], [23], [24], [25]]. While these studies may use disease models, their primary goal is technological advancement rather than therapeutic efficacy.
- **Tissue regeneration (17 %):** This category explored the potential of CMDs for restoring or replacing lost tissue function, distinct from treating specific diseases. This category includes applications such as using CMDs to deliver growth factors or stem cell secretomes for cardiac repair [26], supporting liver regeneration [[27], [28], [29]], or restoring endocrine function [30]. The distinguishing feature of this category is its focus on tissue repair and functional restoration rather than disease treatment.

Our review identified two commercial CMD systems: TheraCyte™, and a hollow-fiber device from CytoTherapeutics. The TheraCyte™ device emerges as the most prevalent system within the reviewed studies, accounting for 28 studies (27 % of all papers). Notably versatile, it has been applied in various contexts, including ovarian, pancreatic, cardiac, and hepatic tissue implantation [26,27,30,31]. With its first appearance in 1999 [32] and continued use in present-day research, the TheraCyte device has established itself as a well-studied and promising platform for CMD therapy. The review also highlights the contributions of CytoTherapeutics (7 studies), a company that specialized in the fabrication of cell encapsulation devices during the late 1990s and early 2000s. The company focused only on nervous system applications, utilizing hollow-fiber shaped polymers and xenogeneic cells [[33], [34], [35], [36]]. However, with the company’s sale in 2000, its contributions end after this time. The remaining majority of the reviewed studies(66 %) explore self-developed and manufactured devices, reflecting the ongoing efforts of numerous research groups to create novel CMD systems.

## 4 Device characteristics

### 4.1 Shape

**Fig. 3:**
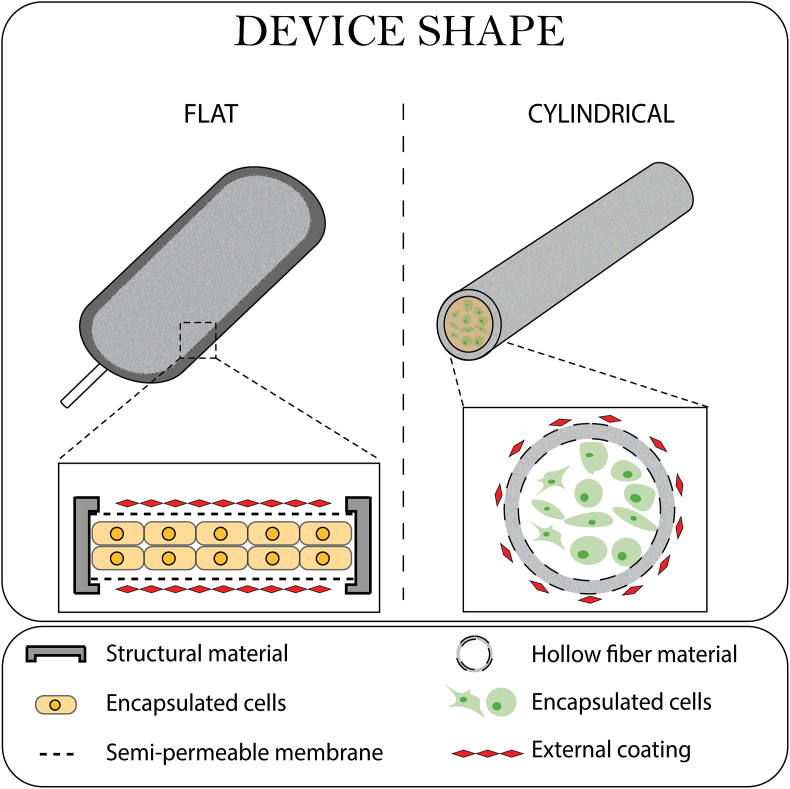
**Device shapes in CMD research.** Illustration of the two primary device shapes found in the studies: flat and cylindrical.

CMDs can be broadly categorized into two main configurations based on their shape during fabrication: flat sheets and cylindrical hollow fibers (Fig. 3). Flat sheets typically feature rectangular or disc-like membranes, with examples such as the TheraCyte devices, which come in three sizes: 4.5 μL (17.5 mm × 7.0 mm × 2 mm), 20.0 μL (22 mm × 11.2 mm × 3 mm), and 40.0 μL (44.2 mm × 11.2 mm × 3 mm) [37]. These designs are optimized for subcutaneous implantation, offering easy handling and retrieval. Cylindrical hollow fibers, on the other hand, are tubular structures with diameters commonly ranging from 0.5 to 1.5 mm and lengths extending from a few centimeters to over 20 cm, depending on the application. These devices excel in maximizing surface area-to-volume ratios, enhancing nutrient and oxygen exchange for encapsulated cells. Our analysis revealed a relatively even distribution between these two designs, with each accounting for roughly 50 % of the studies reviewed.

### 4.2 Material

**Fig. 4:**
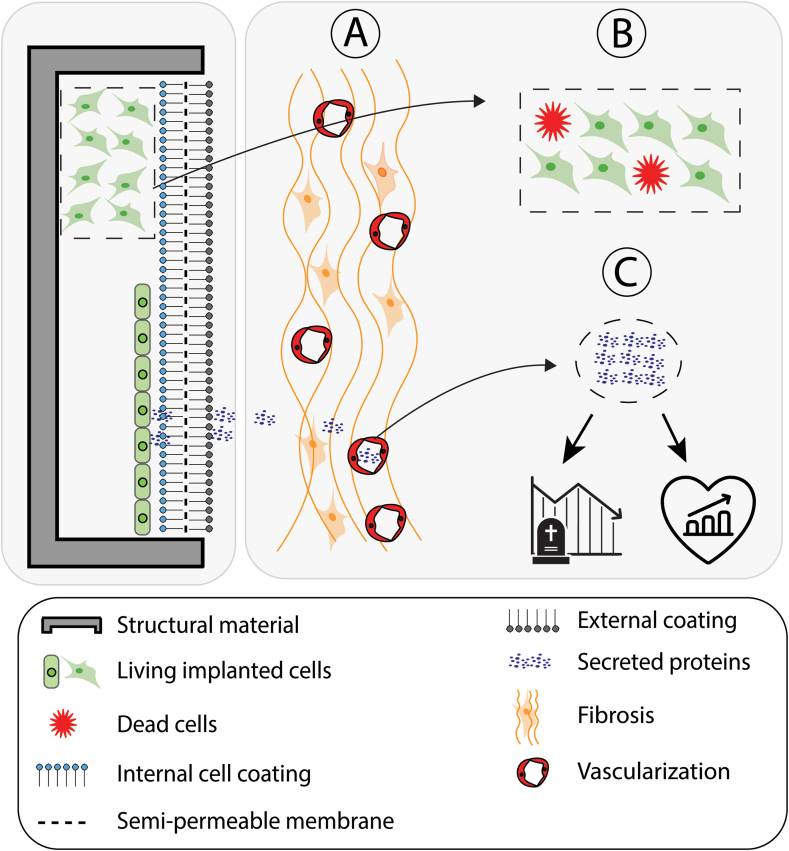
**Device characteristics and readout parameters.** Left: device features such as materials, cells, and coatings. Right: most common readout assays identified in this review: A: Tissue response accommodation, showing fibrotic and vascularizing tissue responses. B: Sustained cellular integrity within the device, focusing on cell viability and differentiation. C: Detection of secreted products in the host’s circulation and their effects on animal mortality and morbidity.

The materials from which a device is built can be distinguished into structure materials, cellular interfaces and biomolecular additives (Fig. 4). Structural materials serve to provide strength and shape the device. The cellular interfaces are intended to efficiently enable communication between the cells in the CMD and the recipient while precluding an immune response or infiltration. Biomolecular additives are specifically designed to direct the cells in the device and the tissue of the recipient to optimal proliferation, differentiation and integration (e.g., growth and differentiation factors or anti-inflammatory agents).

Flat sheet CMDs are typically constructed by adhering two semi-permeable membranes, often made from the same material, to form a thin-film device with a pouch-like structure [[38], [39], [40], [41]]. Alternatively, these membranes can be housed within a supportive framework crafted from structural materials like titanium [25,42], Polydimethylsiloxane (PDMS) [43], or Polyether ether ketone (PEEK) [44]. A notable example of this approach is the TheraCyte device, which utilizes two layers of PTFE membranes encased within an outer polyester mesh, a design principle that has also been adopted in other similar CMDs [20].

In contrast to flat sheets, cylindrical hollow fiber CMDs generally do not require additional support structures. These are typically fabricated through established chemical manufacturing processes, such as the dry-jet wet spinning technique which utilizes a liquid polymer and an annular spinneret [45,46]. Commercially available hollow fiber membranes can also be directly employed for CMD construction [17,47,48]. Additionally, solution electrospinning offers another approach for the fabrication of cylindrical hollow fiber CMDs [49].

The analysis of materials employed in the membrane fabrication revealed a clear dominance of thermoplastics. Polytetrafluoroethylene (PTFE), polycarbonate, poly(ether-sulfone) (PES), and poly(acrylonitrile vinylchloride) (PAV) were the most frequently encountered materials across the reviewed studies. Only two exceptions were identified, where researchers utilized nylon and silicon for membrane construction [50,51] PTFE-based materials emerged as the most prevalent choice, representing approximately one-third of all studies reviewed. Notably, all PTFE-based CMDs adopted a flat sheet configuration. Within this category, the TheraCyte device stands out, accounting for a substantial 82 % of the total. This device features an inner Biopore® membrane with 0.45 μm pores, and an outer membrane with 5 μm pores. Studies exploring self-made devices also adopted similar PTFE-based configurations with two membranes. In contrast to flat sheet devices, hollow fiber CMDs primarily utilize PAV or PES for membrane construction. The studies employing flat sheet configurations typically encapsulated cells directly within standard cell culture media. However, for hollow fiber devices, researchers often opted for an initial encapsulation step using various hydrogel blends such as alginate, collagen, chitosan, and hyaluronate. These hydrogels facilitate nutrient and oxygen diffusion and provide a three-dimensional environment for cells to grow, which improves viability and function within the CMD [52,53].

Biomolecular coatings on CMD surfaces promise better biocompatibility by modulating protein adsorption and initial cellular interactions. For instance, fibronectin/IL-4 coating in PES HF reduced neutrophil accumulation by 71 % and delayed fibrotic capsule formation [54]. THPT coating notably reduced capsule thickness from 150-250 μm to 30–50 μm, downregulated inflammation-related gene expression, and reduced protein content [43]. Additionally, FGF-2 delivery by encapsulated cells increased vessel numbers on the device surface and reduced skin flap necrosis [55]. Other approaches to measure the tissue response are discussed later in this manuscript.

### 4.3 Cell source

The selection of an appropriate cell source for CMDs is a critical decision, influencing device efficacy and long-term functionality. Several key factors guide this selection. Firstly, on-demand availability plays a significant role, with readily available cell sources often prioritized for their practical advantages, facilitating efficient and cost-effective device development. Secondly, the ease and efficiency of cell transfection becomes a consideration if the therapeutic function requires genetic modification. Finally, the specific purpose of the CMD heavily influences the optimal cell source. For instance, while a simple excretory or metabolic function may be adequately supported by xenogeneic cell lines with or without transfection, more intricate therapeutic processes involving complex signaling pathways or species-specific factors may require the use of syngeneic cells [56,57].

Currently, xenogeneic cells, sourced from a different species, dominate the landscape of CMD research, representing 71 % of the studies reviewed (Fig. 2b). Allogeneic cells, originating from the same species but from a different donor, represent another option, constituting approximately 17 % of the studies. When analyzing cell source trends in relation to device types, distinct patterns emerge. Hollow fiber devices predominantly utilize xenogeneic cells (72 %), while allogeneic and multiple cell types each account for 11 % of the studies. In contrast, flat devices exhibit a more diversified distribution, with 55 % employing xenogeneic cells, 21 % allogeneic, and 18 % multiple cell types.

In 31 % of the reviewed studies, researchers employed transfection techniques to induce the expression of target recombinant proteins. This trend also varies with device type: 17 % of studies using hollow fibers employed transfection, while in flat devices, this figure more than doubles, reaching 40 %. These studies predominantly used cell types that do not naturally express the target proteins, such as chinese hamster ovary cells (CHO), fibroblasts, Vascular smooth muscle cells, NTC-200, ARPE-19, HEKepo, and baby hamster kidney (BHK) cells. These cell types were chosen for their robustness, ease of culture and transfection, and high productivity [[58], [59], [60]]. Among these studies, 70 % focused on inducing expression of specific therapeutic proteins such as erythropoietin (EPO), glial cell-derived neurotrophic factor (GNDF), and glucagon-like peptide-1 (GLP-1). EPO was predominantly expressed in myoblasts across various studies [[61], [62], [63], [64]], although other cell types were also employed for this purpose [[65], [66], [67]]. Similarly, GNDF was expressed in different cell types, leading to neuroprotective effects in multiple studies [48,68,69]. Additionally, GLP-1 was targeted for diabetes treatment, with various studies investigating its expression in different cellular contexts [[69], [70], [71]]. The remaining 30 % of transfected studies were used as proof-of-principle applications rather than functional protein secretion, allowing bioluminescence-based assays that simplify viability assessment, a method further elaborated in the read-out section of this manuscript. Interestingly, among all transfected cells, 85 % were xenogeneic, further highlighting their prevalence in CMD research.

## 5 Implantation characteristics

### 5.1 Animal models

Small rodents, predominantly rats and mice, emerge as the predominant animal models utilized in CMD research, encompassing 90 % of the studies reviewed (Fig. 2c). In contrast, larger mammalian species such as sheep, minipigs, and monkeys contribute significantly less, being featured in only 10 % of the included studies. Dogs were involved in two studies [72,73], sheep in three [36,74,75], and non-human primates in four separate investigations [33,[76], [77], [78]]. The use of these larger mammals is mostly associated with the use of CMDs in the nervous system, with the goal of treating neurodegenerative diseases.

### 5.2 Implantation site and duration

**Fig. 5:**
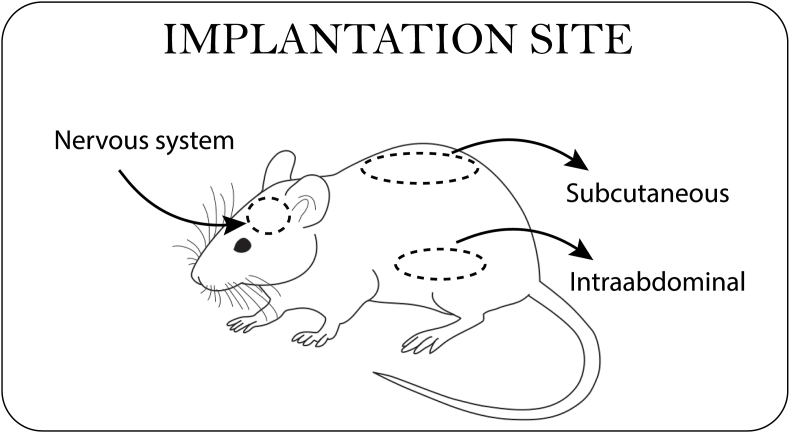
**Implantation sites in animal models used in CMD research.** Illustration of the primary implantation sites used in CMD: nervous system, subcutaneous, and intra-abdominal regions. A rat is used as example animal, but these sites were identified in all animal models.

The selection of an implantation site for CMDs is pivotal, influenced by factors such as device dimensions, shape, experimental feasibility, and therapeutic objectives. Among the most common choices, three primary locations stand out: subcutaneous sites, utilized in 37 % of studies; intraperitoneal locations, in 28 %; and within the nervous system (including the brain and spinal cord), accounting for 25 % (Fig. 5). The remaining 10 % encompass various specific organs or tissues, including the eye, bone, and below the kidney capsule (Fig. 2d).

The choice of implantation site may require a specific CMD shape for optimal integration. Studies employing subcutaneous implantation demonstrate a notable preference for flat devices, with 78 % of these studies utilizing flat devices, predominantly composed of PTFE-based materials such as the TheraCyte. In contrast, intraperitoneal implantation exhibits a more balanced distribution between hollow-fiber cylinders and other flat, but predominantly non-PTFE designs. For nervous system implantation, studies predominantly used cylindrical hollow fiber devices. Except for one instance involving a flat device for spinal cord implantation, all other studies employed hollow fibers made primarily from PAV, or PES.

Optimizing the implantation process can improve device survival and function. One method involves pre-implanting the CMD within the body for a specified period before cell loading. This pre-vascularization step fosters blood vessel growth around the device, establishing a well vascularized microenvironment for encapsulated cells upon final implantation. Studies indicate that pre-implantation for 3 months can alleviate the inflammatory milieu around the final implant site and reduce the number of islets needed for successful diabetes treatment in rodent models [31,79].

In another approach, researchers pre-vascularized CMDs by pre-implanting loaded devices with mesenchymal stem cells or platelet-rich plasma for up to six weeks in mice and non-human primates. After this period, the desired endocrine cells could be injected in the fully vascularized device. This method led to a dose-dependent increase in blood vessel density, up to 270 % compared to unloaded controls, with a promising window for cell transplantation identified at two weeks [80]. Similarly, the incorporation of FTY720, a small molecule with immunomodulatory and pro-angiogenic properties, into electrospun fibers, followed by a 2-week pre-vascularization period before cell addition, enhanced adjacent vasculature around the device compared to unloaded fibers [81].

The site of implantation of the CMD was determined by the purpose of the CMD. Intraperitoneal implantation is mostly used in diabetes treatment (55 %) and liver regeneration (24 %). Nervous system implantations are predominantly aimed at neurodegenerative disease treatment (52 %) and pain relief strategies (16 %). Subcutaneous implantations are also commonly chosen for diabetes treatment (42 %), but also notably when showing proof of concept of a new device (24 %), and treatment of anemia via EPO secretion (13 %).

The distribution of study durations for different implantation sites reveals distinct trends. Intraabdominal implants predominantly feature mid-term studies, with 52 % lasting 1–3 months, and a significant portion (28 %) spanning 0–1 month. Only a small fraction extend beyond 3 months, and none exceed 12 months. Nervous system implants show a focus on short-term studies, with over half (52 %) lasting 0–1 month and a notable portion (32 %) in the 1–3 month range. There are no studies exceeding 6 months in this category. Subcutaneous implants exhibit a more balanced distribution, with 34 % of studies lasting 1–3 months and 29 % covering 0–1 month. A significant portion (18 %) spans 3–6 months, and there is a higher presence of long-term studies (6–12 months) at 13 %, compared to other sites.

## 6 Functional outcomes

### 6.1 The tissue response to the CMD: non-functional fibrotic response

Upon implantation, a series of events known as the fibrotic tissue response (FTR) unfolds around the CMD. In short, plasma proteins and native immune cells like neutrophils and CD3^+^ T-cells adhere to the surface of the implanted material, triggering a cellular response marked by the recruitment of macrophages, which transition from the M1 to the M2 phenotype. This transition is pivotal as it signifies a shift from a pro-inflammatory state (M1) to an anti-inflammatory and tissue-repairing state (M2) [82]. Next, fibroblasts, are drawn to the implant and begin to secrete collagen, the main protein component of scar tissue [83]. Over time, this collagen deposition progressively engulfs the CMD, forming a dense fibrous capsule. This capsule can compromise the function of the implant by hindering nutrient and oxygen diffusion to the encapsulated cells. In severe cases, the lack of oxygen and nutrients leads to hypoxia and necrosis within the implant, ultimately resulting in device failure [84].

Given the critical role of CMD accommodation for functionality, the evaluation of fibrotic tissue response (FTR) is crucial, as demonstrated by its application in the majority (56 %) of the studies. A subset of studies further investigated the FTR process by histologically quantifying the presence of macrophages and CD3^+^ T-cells [17,43,85,86]. Additionally, the upregulation of genes associated with macrophage M1/M2 activation, including tumor necrosis factor-α (TNF-α), interleukin-10, and transforming growth factor-beta 1 (TGF-β1), was quantified in some of these studies using quantitative polymerase chain reaction (qPCR) [17,43,86]. Moreover, the formation of a fibrotic capsule was extensively evaluated across studies, primarily through histological techniques such as hematoxylin and eosin (H&E) staining and collagen-specific stains like Masson’s or Milligan’s trichrome staining. These assessments often included measurements of fibrotic capsule thickness in millimeters, providing insights into the extent of the FTR and its potential impact on CMD performance [[87], [88], [89], [90], [91], [92]].

### 6.2 The tissue response to the CMD: vascularizing response

Angiogenesis is critical for the success of many implantable medical devices, not only to provide the cells in the device with sufficient nutrients and waste removal, but in many instances also because it facilitates the interaction between the milieu interieur of the recipient with the functional cells in the device (e.g insulin production in response to an increase plasma glucose concentration). Surprisingly, only 25 % of studies included an evaluation of blood vessel response. Among these, half relied solely on descriptive histological analysis, primarily using H&E staining to identify adjacent vasculature around the device. The other half employed more detailed vascular analyses, utilizing quantitative measures such as calculating vessel density (total number of vessels per mm^2^ of membrane surface) [14,50,93], determining the percentage area of vascularization through automated counting of red pixels from blood vessels [38], or employing advanced techniques like Laser Doppler [94] or microangiography [55] to combine assessment of vessel density with implant perfusion.

While the FTR and neovascularization are a significant challenge, researchers are actively exploring strategies to mitigate its impact on CMD function. Two broad approaches have emerged: modifying the device itself and optimizing the implantation process. These have been previously discussed in the material and implantation sections. Another innovative approach to increase oxygenation in the device involves a hybrid strategy integrating a slab of photosynthetically active cyanobacteria (*Synechococcus lividus*) on top of an LED light source sandwiched in the CMD, which sustained the viability and function of the implanted islets for up to a month [95].

### 6.3 Functional readouts

Besides the tissue response to the implanted device, the core aspect of CMD research lies in evaluating their functionality. This review identified three major categories of functional readouts employed across the included studies, regardless of the specific therapeutic goal: **Cell viability, Maintenance of functional differentiation, and Generation and impact of cell products.**

### 6.4 Cell viability

A critical factor for successful CMD therapy is the sustained viability of encapsulated cells. Studies utilize various methods to assess cell viability, most of which rely on device explantation. Histology, using techniques like hematoxylin and eosin staining, offers a general evaluation of the device and the surrounding tissue. Some studies incorporate scoring systems to assess the encapsulated cells within the device [42]. Another common approach involves cell viability assays on explanted devices. These assays, such as trypan blue staining for cell viability, the MTT (3-(4, 5-dimethylthiazolyl-2)-2, 5-diphenyltetrazolium bromide) assay for metabolic activity, or the CCK-8 (cell counting kit-8) assay for cell proliferation, provide a snapshot of cell health at the time of retrieval [27,34,96,97] An emerging technique offers the advantage of monitoring cell survival in real-time without device removal, relying on bioluminescence imaging of genetically modified cells. Luciferase is a commonly used enzyme that generates light in the presence of a substrate. By incorporating a luciferase gene into the implanted cells, and injecting the substrate in the surrounding tissue, researchers can monitor their viability and persistence over time through bioluminescence measurements [16,77,80,[98], [99], [100]].

### 6.5 Maintenance of functional differentiation

In addition to ensuring basic cell survival, it is crucial that cells within the CMDs maintain appropriate differentiation to continue providing their functional benefits. Various histological staining techniques are employed to assess cell function and product secretion on explanted devices. Specific histochemical staining targets proteins of interest, evaluating the preserved function of implanted cells. For instance, studies investigating neural implants may use Nissl staining to assess neuron health, acetylcholinesterase staining to evaluate the presence of neurotransmitters essential for muscle movement [21,33,101], or tyrosine hydroxylase, to identify dopamine-containing cells [36,91]. In the case of prostate tissue implants, detection of prostate-specific antigen secreted in the device is essential [22]. Similarly, researchers studying diabetes therapies may employ stains to detect insulin- or glucagon-positive cells within explanted devices [21,[102], [103], [104], [105], [106], [107], [108]]. These histological approaches serve as a bridge to the functional evaluation discussed below.

### 6.6 Generation and impact of cell products

The generation and impact of cell products constitute pivotal aspects of CMD functionality. These devices serve as platforms for the production and release of therapeutic molecules by the encapsulated cells, with hormone secretion being the predominant focus. In studies targeting diabetes treatment, insulin secretion was a primary endpoint, assessed in 80 % of the 36 investigations in this category. Notably, insulin levels increased significantly from 0.12 ± 0.05 ng/ml in diabetic mice to 0.80 ± 0.45 ng/ml post-implantation, reaching levels akin to those in healthy control animals [109]. Similarly, in a canine study, measurable human insulin levels were detected four weeks post-implantation, ranging from 10.5 to 30 mIU/mL [72]. CMDs designed for chronic anemia treatment demonstrated a rapid increase in serum EPO levels by 65.7 ± 12.4 % within 48 h post-implantation [110], while those aimed at growth hormone (GH) secretion maintained elevated human GH levels in the blood, reaching up to 2.5 ng/ml over a six-month period, compared to the absence of this hormone in the control group [111].

Beyond product generation, the ultimate goal of CMD therapy is to induce a beneficial physiological effect in the recipient. This includes assessing downstream impacts of the released products on specific physiological parameters. For instance, in studies targeting diabetes treatment, normoglycemia was achieved in all 36 investigations, with durations ranging from one week to 18 months. In anemia treatment, EPO release significantly increased hematocrit levels, reaching 76.3 ± 2.3 % within three weeks from a baseline of over 20 %. Controlled EPO release led to a 31-fold rise in EPO levels within 48 h, maintaining long-term hematocrit levels of 55 %–70 % for up to 11 months [[112], [113], [114]].

In addition to biochemical and cellular outcomes, morbidity and mortality are critical measures of the intended therapeutic effect of CMD therapy. For example, in neurodegenerative disease treatment, encapsulated GDNF-secreting cells significantly reduced seizures in rats by 93 % [8] and improved brain lesions and forelimb function compared to lesioned rats [7]. Additionally, dosage-dependent neuroprotection was observed with CNTF produced by encapsulated cells [115], while CMDs containing genetically modified xenogeneic cells continuously producing human nerve growth factor(hNGF) attenuated age-related cognitive deficits in aged rats [116]. Similarly, IL-13-secreting cells reduced arthritis severity scores by 50 % [117]. In anti-tumor therapy, endostatin secretion inhibited tumor angiogenesis and decreased tumor size by 50 % in rats over 17 days, despite the eventual loss of encapsulated cells during the study period [14]. In acute liver failure models, transplantation of encapsulated hepatocytes substantially reduced animal mortality from 93 % to 36 % [118], alongside sustained albumin secretion 90 days post-transplantation [119,120].

### 6.7 Trends over time

**Fig. 6:**
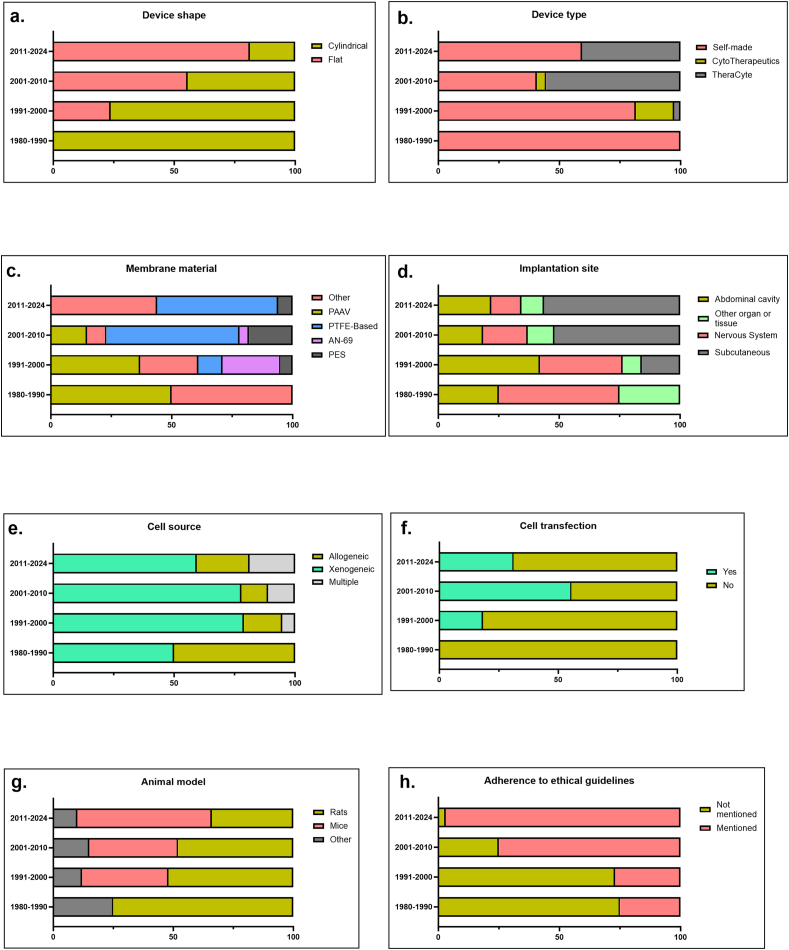
**Decade-wise trends in CMD study characteristics (a**–**h).** Each chart displays the percentage of studies per decade, covering: **(a)** device shape (cylindrical vs. flat), **(b)** device type (self-made, CytoTherapeutics, or TheraCyte), **(c)** membrane material (PAAV, PTFE, AN-69, PES), **(d)** implantation site (abdominal cavity, nervous system, subcutaneous, or other), **(e)** cell source (allogeneic, xenogeneic, or multiple), **(f)** use of transfected cells, **(g)** animal models (mice, rats, or other), and **(h)** adherence to ethical guidelines.

Over the four decades analyzed (1980–2024), cell macroencapsulation devices underwent substantial evolution across multiple parameters (Fig. 6). Device shape showed a dramatic shift from exclusively cylindrical designs in 1980–1990 to predominantly flat configurations (81 %) by 2011–2024. Device manufacturing transitioned from purely self-made devices in the 1980s to a more diverse landscape, with self-made (60 %) and TheraCyte (40 %) devices dominating recent years. Membrane materials evolved significantly, with PAAV declining from 50 % usage in the 1980s to complete disuse by 2011–2024, while PTFE-based materials emerged as a leading choice (50 %) in recent years. Implantation preferences shifted from the nervous system (50 % in 1980–1990) to subcutaneous sites (56 % in 2011–2024). While xenogeneic cell sources remained predominant throughout the study period, their use decreased from 79 % (1991–2000) to 59 % (2011–2024), with increasing diversity in cell source selection. Cell transfection showed variable adoption, peaking at 55 % in 2001–2010. Animal models shifted from primarily rats (75 % in 1980–1990) to mice (56 % in 2011–2024). Adherence to ethical guidelines improved dramatically, from only 25 % of studies reporting guidelines in 1980–1990 to 97 % in recent years, reflecting enhanced research rigor and transparency in the field.

## 7 Discussion

To our knowledge, this systematic review is the first to provide a structured overview of CMD development over the past four decades, offering insights into how these devices have evolved in design, application, and functional outcomes. Our analysis highlights significant shifts in CMD research across this period Initially, CMD applications were limited to encapsulating pituitary cells or xenogeneic whole prostatic tissue in the 1980s [21,22,121]. Over time, the scope of CMD applications has expanded to encompass more than 15 different therapeutic goals. Despite the wide variety of approaches and applications, there are similarities in aspects of CMD design, application and evaluation. This may allow cross-fertilization between research groups focusing on different organ systems.

We observed that technological advances have led to more sophisticated CMD designs. Materials have improved from rather inert hollow fibers to more flat and versatile membranes combined with hydrogels and bioactive coatings. These advancements have enabled devices that better support cell viability, functional differentiation, and interaction with host tissue. Additionally, the choice of cell sources has also changed, with allogeneic cells becoming increasingly prevalent over xenogeneic, and cell transfection techniques being more frequently used to enhance function or facilitate readouts. CMD designs have been optimized for more convenient implantation sites from mainly hollow fibers for intraabdominal implantation to a predominant flat design for mainly subcutaneous implantation. It is important to realize that the use of a particular device design is driven not only by the specific application but also by (commercial) availability. The same applies to the cell types used. An increase in the use of certain cells, materials and designs is thus not necessary a sign of functional superiority.

CMD’s read-out approaches have also evolved over time. The development of more diverse readout systems to assess device functionality has become necessary due to the broadening scope of potential CMD applications. Additionally, greater emphasis has been placed on quantifying the integration of CMDs within the host environment. Detailed analysis of vascularization and fibrosis has become an essential part of evaluation, providing a clearer picture of device integration and longevity. Notably, the data indicates improved adherence to ethical guidelines in more recent CMD research, a critical consideration for the responsible advancement of this technology.

Limitations of the presented studies include the relatively short duration of the experiments and the relatively young age of the experimental animals. Chronic diseases often manifest and progress differently in older patients compared to younger individuals, with factors like immunology, fibrosis, and vascularization playing a significant role [122,123]. The reviewed studies predominantly used young animal models without clearly reporting their ages. However, the target patient population for CMD often suffers from age-related organ failure, necessitating long-term device function. Future studies should therefore incorporate older animal models and extend the evaluation period to better simulate real-world clinical scenarios.

### 7.1 Future directions for CMD research

Based on the findings of our systematic review, recent advancements in biotechnology could provide targeted solutions for the limitations observed in current CMD applications. For instance, while our review highlights a reliance on non-autologous cells, the advent of organoid technology offers the potential to generate patient-specific (autologous) tissues, thus potentially enhancing immune compatibility within the CMD. Additionally, CRISPR-Cas9 technology could rectify genetic abnormalities in organoid cells derived from patients with genetic kidney disorders, supporting the development of personalized therapeutic options in CMDs. Our review also noted a lack of structural complexity in the cellular microenvironment within CMDs; bioprinting could address this by enabling intricate microarchitectures crucial for organ-specific functions like those of the kidney. Finally, synthetic biology approaches could help bridge the gap between biomaterials and living tissue, fostering improved integration and functional longevity in CMD applications.

Taken together, CMDs have a significant future in medical treatments, offering a controllable bridge between extracorporeal bioartificial organs and fully implanted bioengineered organs. Integrating information on various aspects of implantable CMDs can accelerate their development. To advance the field, fostering interdisciplinary collaboration and establishing standardized protocols for CMD production and testing are essential. Long-term *in vivo* studies in older animal models, focusing on device-host tissue interactions, vascularization and fibrotic responses, are crucial. Personalized medicine approaches, with the use of autologous cell sourcing and genetic engineering, can create tailored CMDs, improving efficacy and safety. By addressing these areas, we can bring this promising technology closer to clinical reality.

## Declaration of competing interest

The authors declare that they have no known competing financial interests or personal relationships that could have appeared to influence the work reported in this paper.
