# Supplementary material for: Cell macroencapsulation devices in contemporary research: A systematic review
Source: Regen Ther. 2025 Jun 9;30:144–56. doi: 10.1016/j.reth.2025.05.013 (PMC12180967; doi:10.1016/j.reth.2025.05.013)
Supplement: Multimedia component 1 [file mmc1.docx]

**Supplemental table 1: Search strategy**

PubMed on 27/03/2024

| **#** | **Search syntax** |
| --- | --- |
| 1 | **Encapsulat*[tiab] OR "Cell Encapsulation"[Mesh] or “Macroencapsulation”[tiab]** |
| 2 | **Implant*[tiab] OR "Transplant*"[tiab] OR "Allografts"[Mesh] OR xenograf*[tiab] OR heterograf*[tiab] OR "Heterografts"[Mesh] OR xenotransplan*[tiab] OR heterotransplant*[tiab] OR allograft*[tiab]** |
| 3 | **“Membranes, Artificial"[Mesh] OR (polymer*[tiab] AND membrane*[tiab]) OR TheraCyte[tiab] or (hollow fiber [tiab]) or CytoTherapeutics [tiab] or ViaCyte [tiab]** |
| 4 | **liposome* [tiab] OR nanoparticle* [tiab]** |
| 5 | **#1 AND #2 AND #3** |

Embase on 27/03/2024

| **#** | **Search syntax Embase** |
| --- | --- |
| 1 | **Encapsulat*:ti,ab OR 'cell encapsulation'/exp OR Macroencapsulation:ti,ab** |
| 2 | Implant*:ti,ab OR 'allograft'/exp OR xenograf*:ti,ab OR heterograf*:ti,ab OR 'xenograft'/exp OR xenotransplan*:ti,ab OR heterotransplant*:ti,ab OR allograft*:ti,ab |
| 3 | **'Membranes, Artificial'/exp OR (polymer*:ti, ab AND membrane*:ti,ab) OR TheraCyte:ti,ab OR (hollow fiber:ti,ab)** |
| 4 | **liposome* [tiab] OR nanoparticle* [tiab]** |
| 5 | **#1 AND #2 AND #3** |
| 6 | **#5 NOT #4** |
